# Comparative evaluation of the genomes of three common *Drosophila*-associated bacteria

**DOI:** 10.1242/bio.017673

**Published:** 2016-08-04

**Authors:** Kristina Petkau, David Fast, Aashna Duggal, Edan Foley

**Affiliations:** Department of Medical Microbiology and Immunology, Institute of Virology, University of Alberta, Edmonton AB, T6G 2E1Canada

**Keywords:** *Drosophila*, Intestine, Microbiota, Host-microbe

## Abstract

*Drosophila melanogaster* is an excellent model to explore the molecular exchanges that occur between an animal intestine and associated microbes. Previous studies in *Drosophila* uncovered a sophisticated web of host responses to intestinal bacteria. The outcomes of these responses define critical events in the host, such as the establishment of immune responses, access to nutrients, and the rate of larval development. Despite our steady march towards illuminating the host machinery that responds to bacterial presence in the gut, there are significant gaps in our understanding of the microbial products that influence bacterial association with a fly host. We sequenced and characterized the genomes of three common *Drosophila*-associated microbes: *Lactobacillus plantarum*, *Lactobacillus brevis* and *Acetobacter pasteurianus*. For each species, we compared the genomes of *Drosophila*-associated strains to the genomes of strains isolated from alternative sources. We found that environmental *Lactobacillus* strains readily associated with adult *Drosophila* and were similar to fly isolates in terms of genome organization. In contrast, we identified a strain of *A. pasteurianus* that apparently fails to associate with adult *Drosophila* due to an inability to grow on fly nutrient food. Comparisons between association competent and incompetent *A. pasteurianus* strains identified a short list of candidate genes that may contribute to survival on fly medium. Many of the gene products unique to fly-associated strains have established roles in the stabilization of host-microbe interactions. These data add to a growing body of literature that examines the microbial perspective of host-microbe relationships.

## Abstract (precis)

**Summary:** We examined the genomes of *Drosophila*-associated bacteria to identify factors that allow survival within the host. These preliminary studies may point at bacterial products that influence host health.

## INTRODUCTION

Environmental, microbial, and host factors act at mucosal barriers to establish a unique microclimate that shapes the lives of all participant species (Spor et al., 2011). For example, expression of a host genotype in gastrointestinal tissues works in concert with extrinsic factors to determine microbial associations (Donaldson et al., 2015). The metabolic outputs of the gastrointestinal microbiota influence critical events in the host such as education of immune phenotypes (Hooper et al., 2012; Round and Mazmanian, 2009), development of intestinal structures (Kamada et al., 2013), and access to essential micronutrients (Hacquard et al., 2015). Given the intertwined relationship between host phenotype and microbial genotype, it is of some surprise that hosts often tolerate extensive alterations to their microbiota in response to environmental shifts, such as changes in diet (David et al., 2014). However, alterations to the gastrointestinal microbiota are not invariably without consequence, and intestinal dysbiosis may lead to chronic, debilitating, and occasionally deadly diseases within the host (Belkaid and Hand, 2014; Lee et al., 2011; Schwabe and Jobin, 2013; Wen et al., 2008; Wu et al., 2010). Our appreciation of the holobiont as an intricate network of biochemical and genetic transactions between multiple participants mandates a thorough evaluation of the microbial genomes that shape host physiology. Unfortunately, such studies are tremendously complex in conventional mammalian models due to the size of the microbiome, and also the lack of laboratory techniques for the isolation and manipulation of many mammalian commensals.

The simple invertebrate *Drosophila melanogaster* is an excellent model holobiont (Buchon et al., 2013; Ma et al., 2015). From a developmental perspective, the *Drosophila* posterior midgut shares a number of important similarities with the small intestine of more complex mammalian counterparts (Buchon et al., 2013). Both organs are endodermal in origin, and are surrounded by a sheath of mesodermal visceral muscle (Spence et al., 2011; Tepass and Hartenstein, 1994). The mammalian small intestine and *Drosophila* posterior midgut are maintained by regularly spaced, basal intestinal stem cells that generate transitory progenitor cells (Barker et al., 2008; Jiang and Edgar, 2012; Takashima and Hartenstein, 2012); the non-proliferative enteroblasts of *Drosophila*; and the transient-amplifying cells of mammals. In both systems, signals along the Notch-Delta axis promote differentiation of transitory progenitors into secretory enteroendocrine cells or absorptive enterocytes (Buchon et al., 2013; Peterson and Artis, 2014). In contrast to mammals, *Drosophila* lacks specialized basal paneth cells for the release of antimicrobial peptides. Nonetheless, the fly genome encodes antimicrobial peptides that actively contribute to the control of intestinal symbionts and pathogens (Ryu et al., 2008), indicating the release of such factors into the *Drosophila* intestinal lumen. In both the mammalian small intestine and *Drosophila* midgut, host factors and biogeography favor association with members of the *Lactobacillaceae* family (Donaldson et al., 2015; Matos and Leulier, 2014). In return, metabolites from *Lactobacilli* activate host response pathways that promote intestinal stem cell proliferation and reactive oxygen species generation (Jones et al., 2013). Combined with the genetic accessibility of flies and their suitability for longitudinal studies of large populations in carefully defined environments, these attributes establish *Drosophila* as an excellent system to decipher the forces that determine genetic interactions within a holobiont (Buchon et al., 2013; Charroux and Royet, 2012; Ferrandon, 2013).

In contrast to conventional vertebrate models, the *Drosophila* microbiome consists of a small number of aerotolerant bacterial species that are easily isolated and cultured (Broderick and Lemaitre, 2012). The adult *Drosophila* intestine hosts little to no bacteria immediately after emergence from the pupal case and the microbiotal population grows in number over time (Clark et al., 2015). Several studies established that environmental factors and host genotype influence the diversity of the microbiota (Chandler et al., 2011; Ryu et al., 2008; Wong et al., 2011). It is unclear if bacteria establish stable associations with the host gut, or if they cycle from the intestine to the environment and back (Blum et al., 2013; Broderick et al., 2014). Nonetheless, lab-raised and wild *Drosophila* frequently associate with representatives of the genii *Lactobacillus* and *Acetobacter*. These data suggest that the intestinal lumen of an adult fly favors the survival of specific bacteria, and that such bacteria encode the necessary factors to survive or proliferate within a *Drosophila* intestine.

Consistent with a long-term association between the fly intestine and specific microbes, many *Drosophila* phenotypes are influenced by individual *Lactobacillus* or *Acetobacter* species. For example, several strains of *Lactobacillus plantarum*, a common *Drosophila*-associated microbe, promotes larval development via regulation of the TOR signal transduction pathway and induction of intestinal peptidases (Erkosar et al., 2015; Storelli et al., 2011), while *Acetobacter pomorum* regulates host insulin growth factor signals to promote development and metabolic homeostasis (Shin et al., 2011). In addition, members of the *Acetobacter* and *Lactobacillus* populations regulate levels of essential nutrients in the host (Chaston et al., 2015; Huang and Douglas, 2015; Wong et al., 2014). Combined, these data present a compelling argument that *Lactobacilli* and *Acetobacter* are important members of the *Drosophila*-microbe holobiont.

Despite our advances in the elucidation of *Lactobacillus* and *Acetobacter* influences on their *Drosophila* host, it is unclear if the individual species encode factors that permit survival during passage through the adult *Drosophila* intestine. We prepared whole genome sequences of three bacterial species that regularly associate with *Drosophila* – *Lactobacillus brevis*, *Lactobacillus plantarum*, and *Acetobacter pasteurianus*. These sequences included those for a *Lactobacillus plantarum* strain isolated from our lab-raised flies, and a separate strain isolated from a wild *Drosophila melanogaster.* For each species, we compared *Drosophila*-associated bacterial genomes, including ones reported previously, to the genomes of reference strains isolated from non-*Drosophila* sources. We noted few differences between the genomes of environmental and *Drosophila* associated *Lactobacillus* species, and found that environmental *Lactobacilli* readily established stable associations with a *Drosophila* host. In contrast, we identified an *A. pasteurianus* strain that apparently fails to associate with adult *Drosophila*. In follow-up work, we showed that this particular strain does not survive culture on conventional fly food. Comparisons between the association-competent and incompetent strains of *A. pasteurianus* uncovered a short list of possible regulators of *A. pasteurianus* viability on fly food.

## RESULTS

The intestine contains structural and chemical barriers that typically inhibit bacterial growth or viability. In response, the intestinal microbiota express factors that overcome host defenses to permit bacterial survival. Here, we examined the genomes of *L. brevis*, *L. plantarum* and *A. pasteurianus*, which are all common members of the *Drosophila* intestinal community. For each species, we studied whole-genome sequences of bacterial strains that we isolated from adult *Drosophila* intestines, and compared them to related strains isolated from the environment. Details on the respective genomes characterized in this study are presented in Table 1.

**Table 1.**
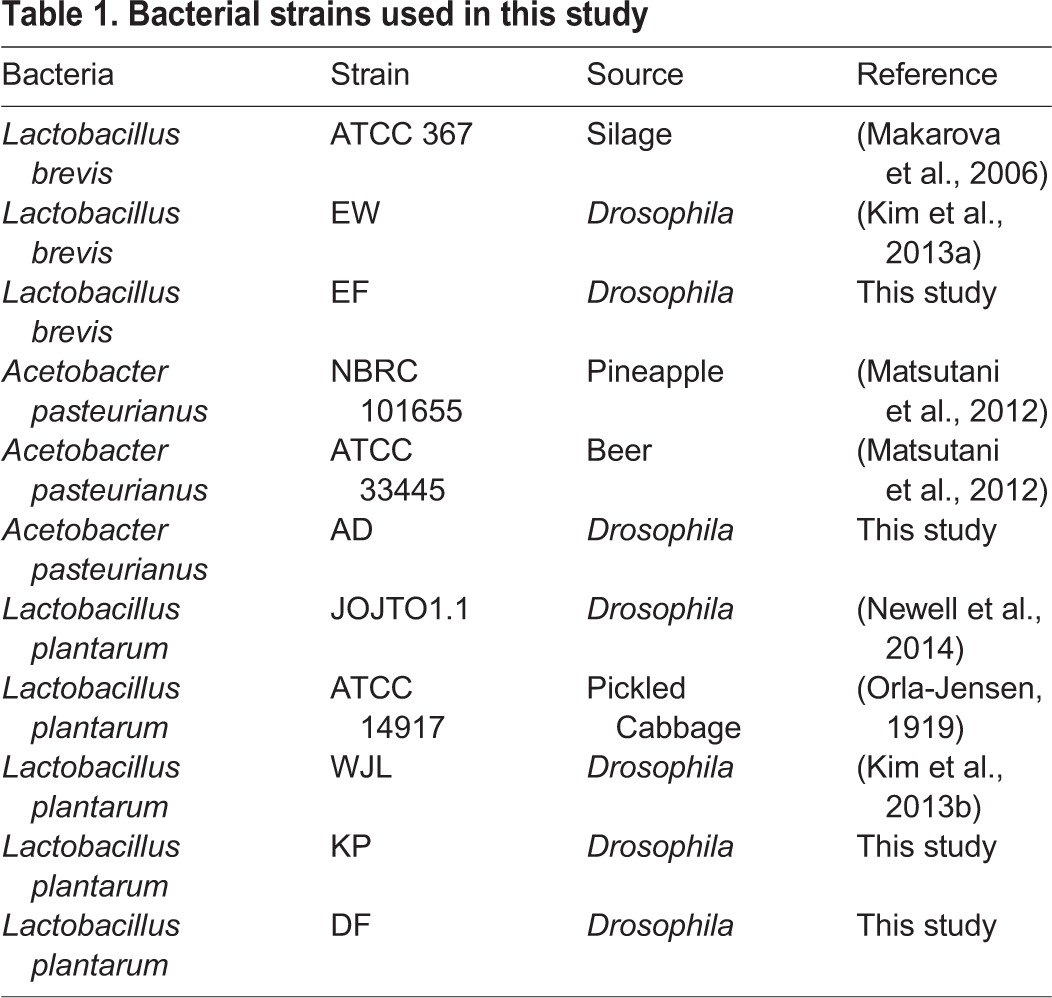
**Bacterial strains used in this study**

We processed each genome in a similar manner. Where necessary, we used genomic databases to identify the bacterial species of newly sequenced genomes. We then annotated each genome with RAST, used PHAST to scan each genome for intact prophages, and searched for possible CRISPR arrays in the respective genomes. We scrutinized the annotated genomes for functions that might facilitate microbial survival within the intestinal lumen, with a focus on genes involved in signal transduction, transcriptional responses, orchestration of stress responses, or induction of virulence factors. Finally, we compared environmental and *Drosophila*-associated genomes for each species to identify bacterial factors that are unique to *Drosophila*-associated genomes. We present the results for each genus below.

### *Lactobacillus brevis* and *Lactobacillus plantarum*

#### General genomic features

**Fig. 1.**
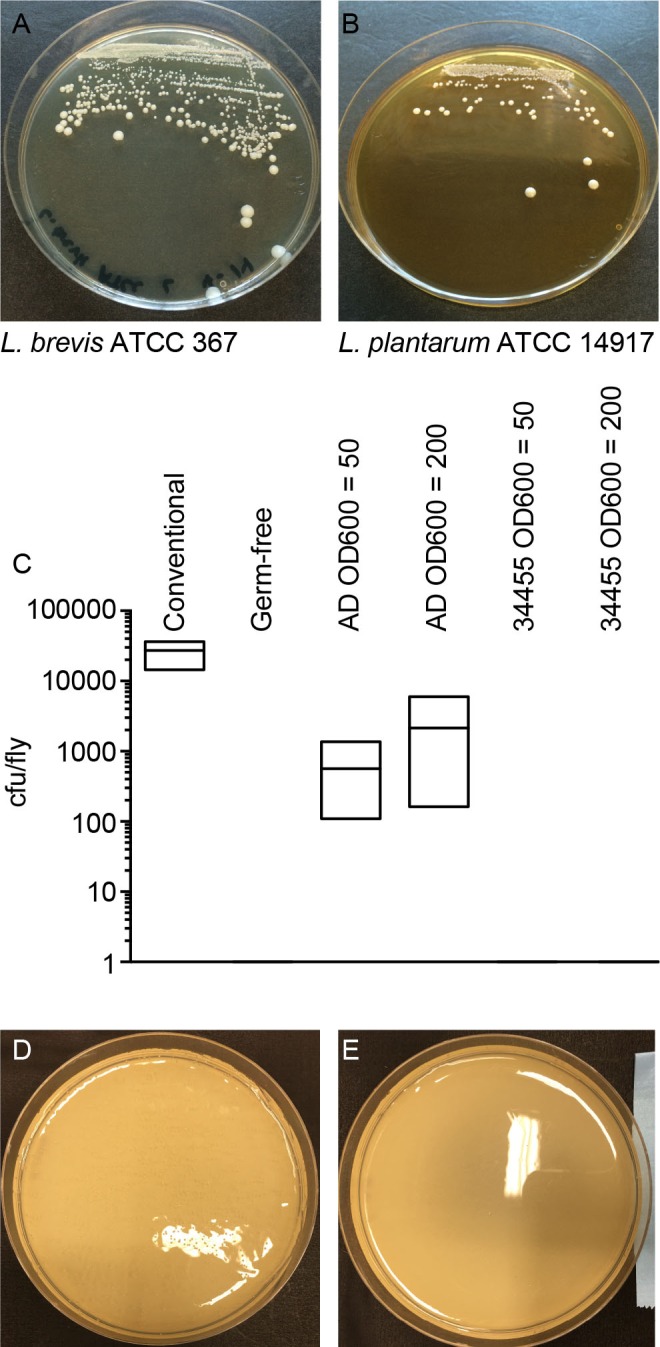
**Evaluation of bacterial strain survival.** **(**A,B) Homogenates from gnotobiotic flies mono-associated with *L. brevis* ATCC 367 (A) and *L. plantarum* ATCC 14917 (B), 10 days after the initial feeding. Each plate contains the equivalent of 1% of the homogenate of an entire fly. (C) Quantification of *A. pasteurianus* association with conventionally reared (column 1) flies, germ-free (column 2) flies, gnotobiotic flies that were fed *A. pasteurianus* strain AD at OD600 of 50 and 200, respectively (columns 3 and 4), or gnotobiotic flies that were fed *A. pasteurianus* strain ATCC 33445 at OD600 of 50 and 200, respectively (columns 5 and 6). Each column shows the results of three separate measurements, and association was measure as bacterial colony-forming units per fly. (D, E) Liquid cultures *A. pasteurianus* AD (D) and *A. pasteurianus* ATCC 33445 (E) were added to fly food, incubated at 29°C for 1 week, rinsed in MRS and re-plated on selective plates.

*L. brevis* is a common member of the *Drosophila* intestinal microbiota, and the whole genome sequence of a fly-associated strain, *L. brevis* EW is available (Kim et al., 2013a). We prepared a whole-genome sequence of an additional *L. brevis* strain (*L. brevis* EF) that we isolated from the intestines of wild-type adult *Drosophila* from our lab. For comparative purposes, we extended our study to include the genome of the environmental ATCC 367 strain. We plated homogenates from flies ten days after feeding a mono-culture of the ATCC 367 isolate and found that *L. brevis* ATCC 367 retained an association with wild-type adult *Drosophila*, confirming that the ATCC 367 strain is association-competent (Fig. 1A). Genome-to-genome distance calculations suggest that the *Drosophila*-associated EW and EF strains are more closely related to each other than to the ATCC 367 strain (Table 2). The genomes of *Drosophila*-associated strains are also larger than the environmental strain, with approximately 500,000 nucleotides more, and an additional 500 coding sequences (Table 2).

**Table 2.**
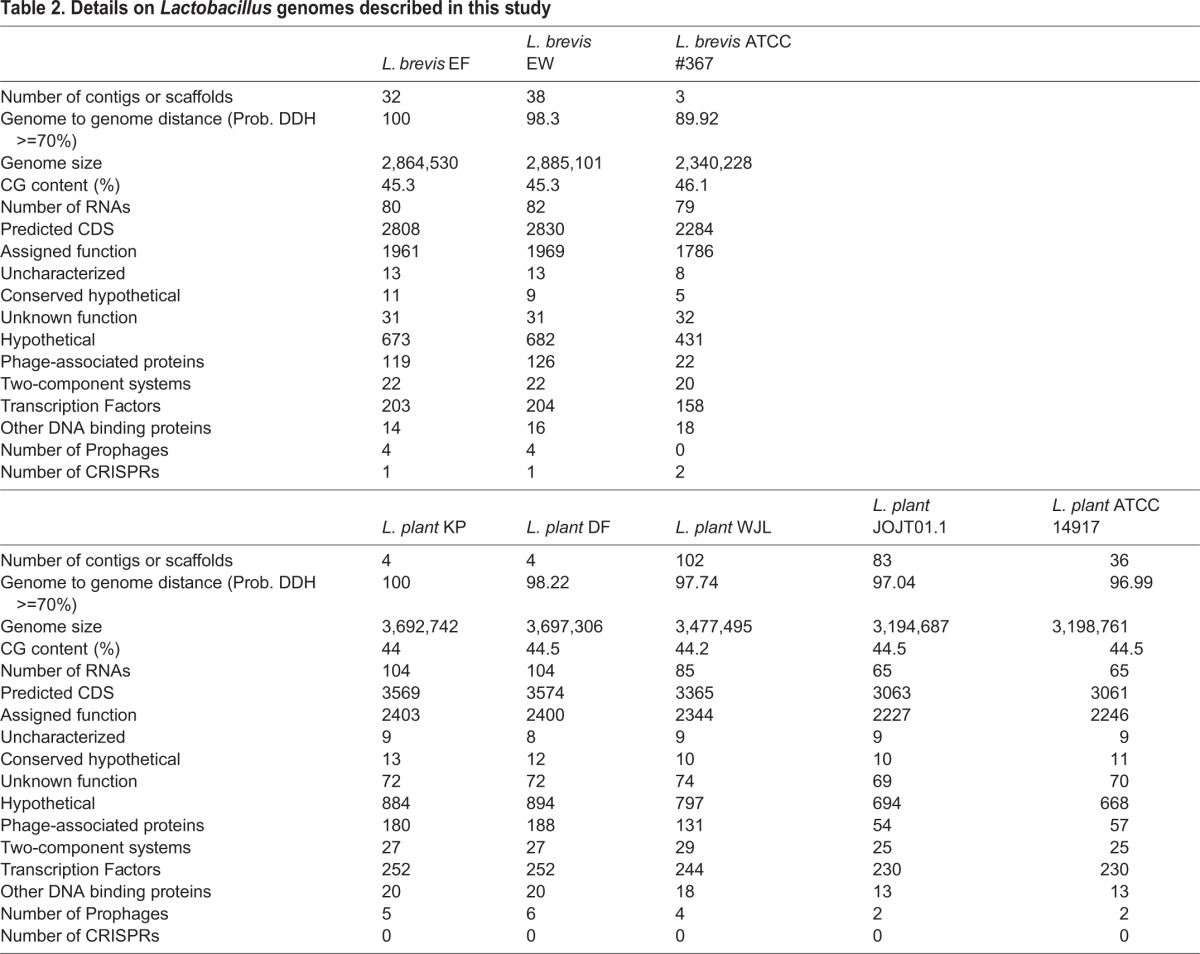
**Details on *Lactobacillus* genomes described in this study**

**Fig. 2.**
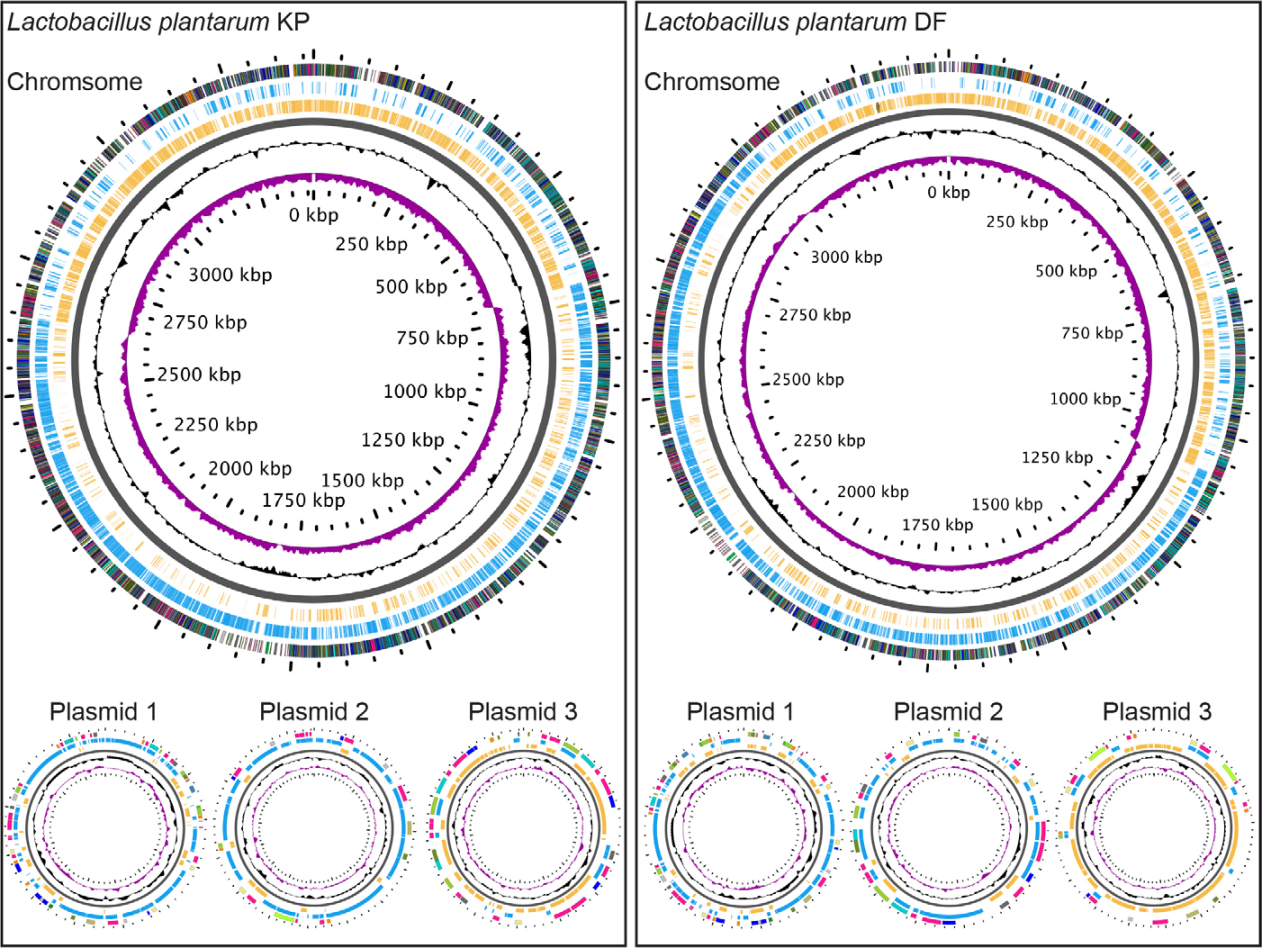
**Illustrations of the genomes for *L. plantarum* strains KP and DF.** GC skew is indicated in purple, and GC content is indicated in black. All positive strand ORFs are shown in blue, and negative strand ORFs are shown in yellow.

For comparative studies of *L. plantarum*, we focused on the environmental ATCC 14917 strain. *L. plantarum* ATCC 14917 was isolated from pickled cabbage. Similar to the ATCC 367 strain of *L. brevis*, we noticed that the ATCC 14917 strain of *L. plantarum* remained associated with wild-type *Drosophila* ten days after feeding (Fig. 1B). We compared the ATCC 14917 strain to four *Drosophila*-associated genomes: WJL, DMCS_001, DF and KP. WJL and DMCS_001 were isolated from *Drosophila* raised in geographically separate labs (Kim et al., 2013b; Newell et al., 2014). We isolated the KP strain from the intestines of our lab-raised wild-type strain, and the DF strain from an isofemale wild *Drosophila melanogaster* line that we captured in Edmonton, Canada in the summer of 2014. The DF and KP genomes encode one chromosome and three closely related plasmids each (Fig. 2). While all five genomes are closely related, genome-to-genome distance calculators suggest a greater degree of identity among the *Drosophila*-associated KP, DF, WJL and DMCS_001 strains (Table 2). In general, the environmental genome is smaller than the *Drosophila*-associated genomes, encodes fewer RNAs and coding sequences, and contains fewer phage-associated proteins (Table 2).

#### Environmental response factors

We then examined genetic regulatory networks within the individual *Lactobacillus* strains to determine if *Drosophila*-associated strains encode distinct regulatory components that permit adaptation to the harsh environment of an adult intestine. For these studies, we paid particular attention to two-component systems, transcription factors and additional DNA-binding proteins within the respective genomes. We did not observe substantial differences between *Drosophila*-associated and environmental genomes for either *L. brevis* or *L. plantarum* (Table 2). Likewise, we only observed slight differences between *Drosophila*-associated and environmental strains when we considered genes dedicated to signal transduction, stress responses, or virulence (Table 2).

#### Prophages and CRISPR responses

Comparisons between environmental and *Drosophila*-associated *Lactobacillus* genomes uncovered a propensity for prophage accumulation within the *Drosophila*-associated genomes. For example, we detected an average of four intact prophage genomes in *Lactobacillus* strains isolated from flies, and a maximum of two prophage genomes in environmental strains. The EW and EF *L. brevis* genomes include four intact temperate prophages, compared to an absence of prophages from the environmental *L. brevis* strain (Table 2). We found CRISPR sequences that target a common *Lactobacillus* phage within all three genomes, while the environmental strain encoded a separate CRISPR array that targets a *Lactobacillus* plasmid (Table 2). These results suggest an ongoing interaction between prophages and CRISPR defenses in the genomes of *Drosophila*-associated *L. brevis* strains. Similar to our observations with *L. brevis* genomes, we observed a greater number of intact prophage genomes in *Drosophila-*associated *L. plantarum* strains than in the environmental strain (Table 2). The main difference between the *Drosophila*-associated *brevis* and *plantarum* strains is that the *plantarum* strains do not appear to encode CRISPR-dependent anti-phage defenses within their genomes.

#### Function-based comparisons of *Drosophila*-associated and environmental *Lactobacillus* strains

In general, the data above suggest very minor differences between the genomes of *Drosophila*-associated and environmental strains of *Lactobacilli*. To characterize these differences in greater detail, we performed a function-based comparison of the 185 genes that are common to *Drosophila*-associated *L. brevis* genomes, but absent from the environmental strain. This set of 185 genes describes thirteen distinct functional categories, with forty-seven unique roles (Table 2). Unsurprisingly, phage and CRISPR-associated gene products account for two of those categories, and cover eleven of the forty-seven unique roles.

**Table 3.**
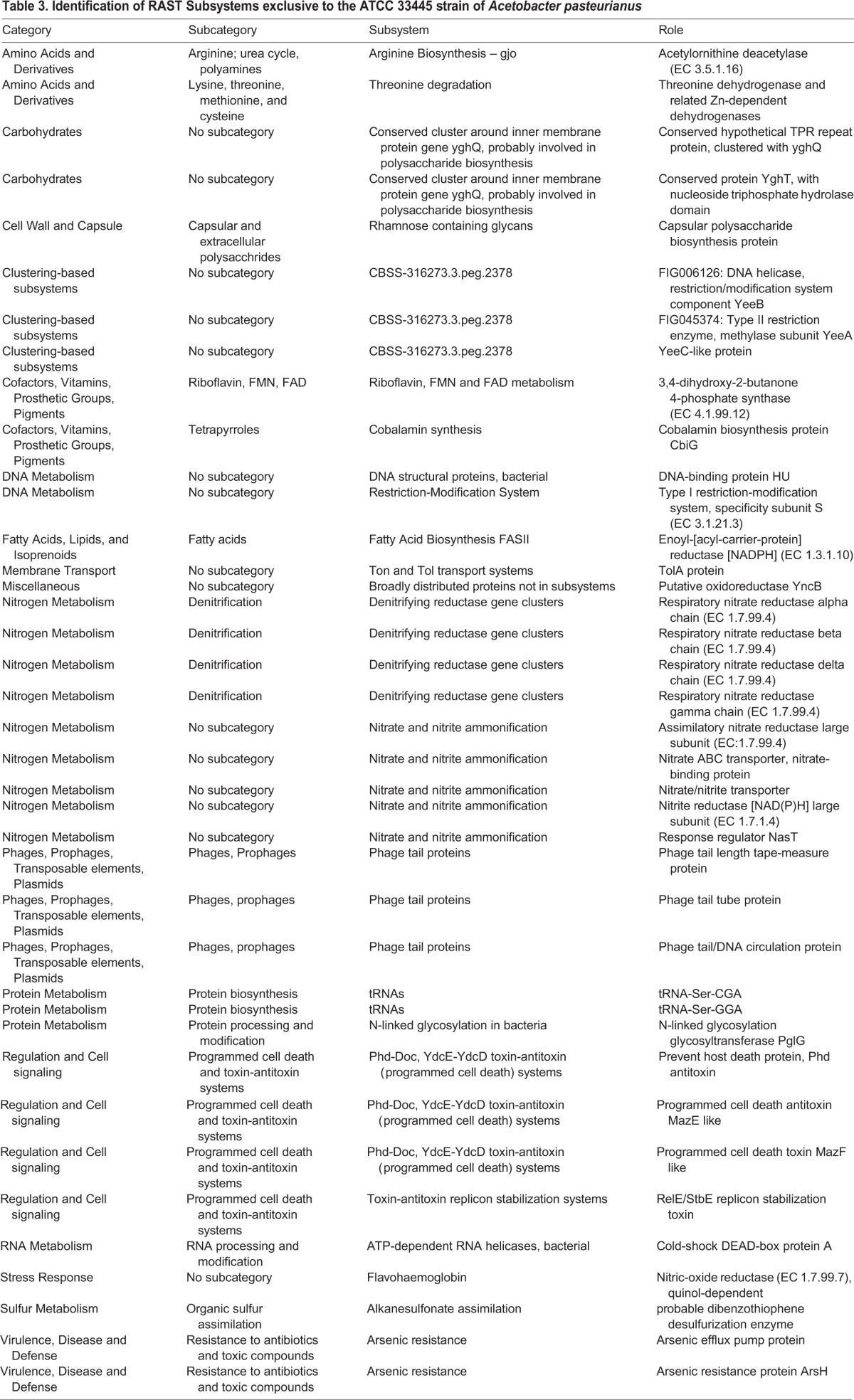
**Identification of RAST Subsystems exclusive to the ATCC 33445 strain of *Acetobacter pasteurianus***

Of the remaining gene products, the dominant functional categories are dedicated to roles that appear suited for survival within an intestine. These include a biochemical cascade that converts α-D-glucose-1-phosphate to dTDP-4-dehydro-6-deoxy-L-mannose, an exopolysaccharide that contributes to prokaryotic survival within a host intestine (Ruas-Madiedo et al., 2006; Zivkovic et al., 2015), and gene products that contribute to the formation of rhamnose-containing glycans, a cell membrane component of acid-fast bacteria that affects several host-microbe interactions, such as adhesion, recognition, and biofilm formation (Martinez et al., 2012).

We also identified gene products within *Drosophila*-associated *L. brevis* genomes that facilitate nutrient acquisition from different sources. Bacteria frequently respond to limitations in nutritional environments through activation of the cAMP receptor protein, a transcription factor that we did not identify in the environmental strain of *L. brevis*, but found in both *Drosophila*-associated strains. The cAMP receptor protein controls, among other things, the expression of gene products that coordinate metabolism of citrate (Meyer et al., 2001), a function that is also enriched among associated *Drosophila*-associated *L. brevis* genomes. In lactic acid bacteria, citrate lyase is activated in acidic environments such as those found in the gut, and increases carbon utilization and energy generation by blocking the inhibitory effects of the *Lactobacillus* fermentation product lactate (Magni et al., 1999). Finally, we detected an enrichment of genes involved in the transport and degradation of pectin in *Drosophila*-associated *L. brevis* genomes. Pectin is an abundant source of energy and carbon for bacteria that grow on plant and vegetable surfaces, and microbial consumption of pectin accelerates the decay of organic matter.

When we looked at the thirty-five genes exclusively observed in the genomes of DF, KP, WJL and DMCS_001 *L. plantarum* strains, the majority (nineteen) were prophage genes, and an additional five were hypothetical proteins of unknown function. Rather strikingly, several of the remaining genes encode products that actively suppress the growth of competing microbes. These include the *PlnMNO* operon that encodes a bacteriocin and cognate immunity protein (Diep et al., 1996), and 1,3-propanediol dehydrogenase, an enzyme that converts propane-1,3,-diol to 3-hydroxypropanal. 3-hydroxypropanal, also known as reuterin, is a *Lactobacillus reuteri* metabolite that exerts broad-spectrum microbicidal effects on intestinal microbes in other animals (Jones and Versalovic, 2009).

### Acetobacter pasteurianus

#### General genomic features

Although *Acetobacter* frequently associate with *Drosophila* in the wild and in the lab, we are unaware of any whole-genome sequences of *A. pasteurianus* strains derived from the intestines of adult *Drosophila*. To address this shortcoming, we completed a whole-genome sequence of an *A. pasteurianus* strain (*A. pasteurianus* AD) that we isolated from the intestines of wild-type *Drosophila*. For comparative purposes, we examined the available genomic sequences of the NBRC 101655 strain, and the ATCC 33445 strain. Our initial attempts to generate gnotobiotic flies, suggested that the ATCC 33445 strain fails to associate with *Drosophila*, something we subsequently confirmed (Fig. 1C). These data suggest that the ATCC 33445 isolate is either incapable of survival within the fly gut, or incapable of growth on fly culture medium. To distinguish between these possibilities, we examined the viability of the ATCC 33445 isolate on fly food in the absence of *Drosophila*. The AD strain isolated from *Drosophila* survives culture on fly food (Fig. 1D), however, we found that the ATCC 33445 strain failed to do so (Fig. 1E).

**Fig. 3.**
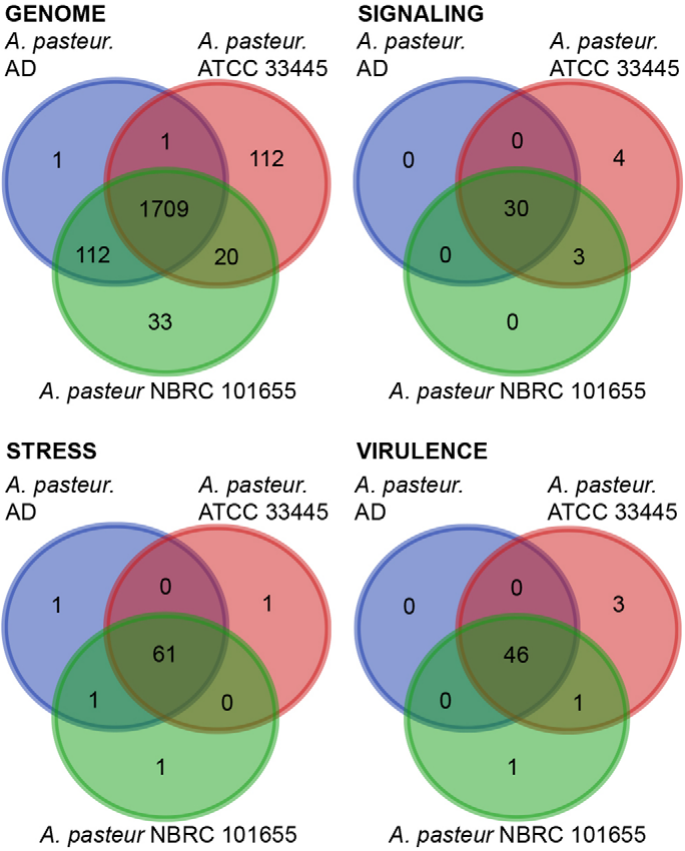
**Distribution of unique gene functions in the genomes of *A. pasteurianus* strains AD, ATCC 33445 and NBRC 101655.** All data are based on gene function annotations within RAST and exclude gene products with unknown functions.

The different viability profiles of the different strains prompted us to compare the AD, NBRC 101655, and ATCC 33445 genomes. At first glance, we did not observe substantial differences between the respective genomes. Each genome is approximately 3 MB in length, with similar GC content and similar numbers of RNA, and predicted coding sequences (Table 4). From an evolutionary perspective, *A. pasteurianus* AD appears more closely related to the NBRC 101655 strain than the ATCC 33445 strain (Table 4). Consistent with a greater evolutionary distance to the ATCC strain, we found that the ATCC 33445 genome encodes 112 unique proteins, while the NBRC 101655 and AD strains share 112 genes that are absent from the ATCC 33445 genome (Fig. 3).

**Table 4.**
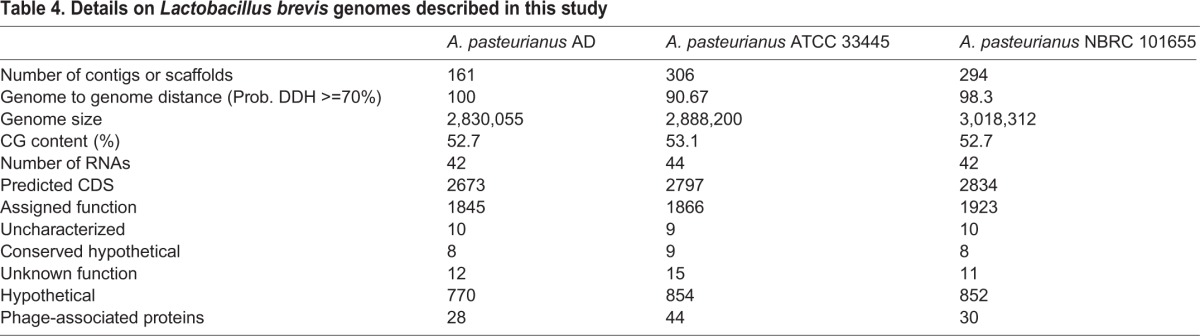
**Details on *Lactobacillus brevis* genomes described in this study**

#### Environmental response factors

As with *L. brevis*, we first compared the *Drosophila*-associated and environmental genomes for distinctions in gene products that respond to environmental factors. Specifically, we looked at signaling factors stress response factors, and virulence factors (Fig. 3). Across this series of comparisons, the most pronounced differences were commensurate with a closer relationship of the AD strain to the NBRC 101655 strain than to the ATCC 33445 strain. Thus, this admittedly limited comparison does not appear to identify genomic components that readily distinguish *Drosophila*-associated *A. pasteurianus* genomes from environmental counterparts. Nonetheless, these functional characterizations uncover differences between the AD and NBRC 101655 *A. pasteurianus* genomes that survive passage on *Drosophila* medium, and the ATCC 33445 genome that fails to do so.

#### Function-based comparisons of individual strains of *Acetobacter pasteurianus*

Our fortuitous identification of an environmental *A. pasteurianus* strain that fails to grow on fly food under experimental conditions that permit growth of all other *Lactobacillus* and *Acetobacter* strains tested allowed us to explore *A. pasteurianus* genomes for factors that may permit survival within a *Drosophila*-friendly environment. We reasoned that the AD and NBRC 101655 genomes encode biochemical functions absent from ATCC 33445 that permit survival on fly food, or that the ATCC 33445 genome encodes biochemical functions absent from the other strains that prevent survival on fly food. This prompted us to identify biological subsystems shared exclusively by the AD and NBRC 101655 (Table 5), or unique to the ATCC 33445 genome (Table 3).

**Table 5.**
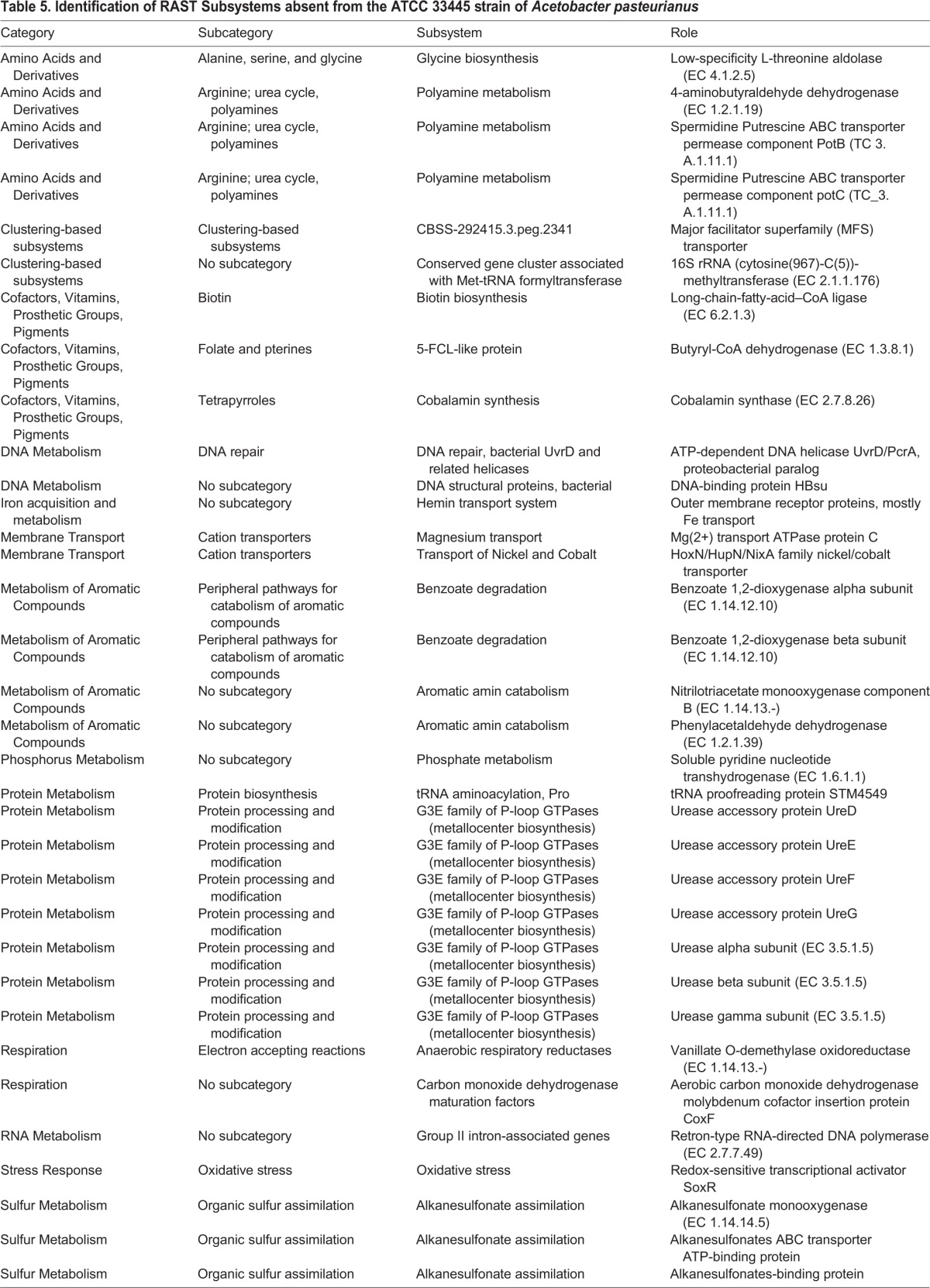
**Identification of RAST Subsystems absent from the ATCC 33445 strain of *Acetobacter pasteurianus***

In this comparative analysis, we noted four subsystems exclusive to the AD and NBCR 101655 genomes that may explain their ability to survive on fly food. Both strains encode polyamine metabolism factors that are frequently associated with cellular growth and survival, and have established roles in the formation of biofilms (Di Martino et al., 2013). The association-competent genomes also encode factors necessary for the conversion of urea to ammonium and carbon dioxide. A similar system operates in *Helicobacter pylori* where it raises the gastric pH to generate a more hospitable environment for microbial survival (Scott et al., 1998). We also detected the redox-sensitive transcriptional activator SoxR in both association-competent genomes. SoxR promotes microbial survival by countering the antibacterial actions of superoxide anions (Imlay, 2015). Finally, we detected several genes that contribute to organic sulfur assimilation in association-competent genomes. These gene products may allow *A. pasteurianus* AD and NBRC 101655 to use alkanesulfonates as a source of sulfur during sulfate or cysteine starvation and may provide both strains a competitive advantage if sulfur is limiting.

The association-incompetent ATCC 33445 strain also encodes products that may contribute to generation of ammonia. However, the ATCC 33445 strain apparently relies on respiratory nitrate reductase and nitrite reductase to generate ammonia, as well as assimilatory nitrate reductase to access nitrate for metabolic growth. This represents an entirely different strategy to use nitrogen as a fuel for metabolic energy and growth. We also observed two toxin-antitoxin systems unique to the association-incompetent ATCC 33445 genome – an addiction module toxin that ensures propagation of plasmids to progeny cells (Engelberg-Kulka and Glaser, 1999), and a MazE/MazF type toxin-antitoxin (Masuda et al., 1993). The MazE/MazF system induces programmed cell death in prokaryotic cells in response to stressful environments.

## DISCUSSION

The last decade witnessed a proliferation of elegant studies that uncovered critical host responses to microbial factors in the *Drosophila* intestine [reviewed in Buchon et al. (2013)]. Bacterial cues promote larval growth (Shin et al., 2011; Storelli et al., 2011), direct innate immune responses (Broderick et al., 2014; Erkosar et al., 2014), orchestrate the proliferation of intestinal stem cells (Buchon et al., 2009a,b), and regulate the uptake and storage of nutrients (Wong et al., 2014). Despite the importance of the intestinal microbiota for *Drosophila* health and development, there are gaps in our understanding of the biochemical events that permit bacterial survival within the hostile terrain of a fly intestine. Recent studies identified microbial metabolism and stress response pathways that mediate interactions between intestinal bacterial and their *Drosophila* host (Chaston et al., 2014; Newell et al., 2014). In this study, we examined the genomes of *Drosophila*-associated strains of *L. brevis*, *L. plantarum*, and *A. pasteurianus*. We were particularly interested in the identification of candidate bacterial factors that could permit survival within the intestines of adult flies. To this end, we compared fly-associated genomes to environmental strains of the same species. For each species, we observed a small number of genetic pathways that were exclusive to the *Drosophila*-associated genomes. Many of the *Drosophila*-associated pathways encode products with established roles in host-microbe interactions, raising the possibility these products may facilitate association of *Drosophila* with the individual strains.

### Caveats

Interpretation of the data presented in this study should be influenced by several important caveats. The experimental design in this study does not distinguish between true colonization of an adult intestine and simple passage through the gut. To date, there are no studies that have identified *Lactobacillus* strains that fail to associate with the adult intestine of *Drosophila*. We also observed that environmental strains of *L. brevis* and *L.* plantarum form stable associations with *Drosophila*. The rather indiscriminate associations between flies and *Lactobacilli* confound attempts to identify fly-specific response factors within a bacterial genome. Indeed, it cannot be excluded that core elements of *Lactobacillus* genomes are sufficient for survival during transit through a fly intestine. In contrast, we have identified an *A. pasteurianus* strain that appears incapable of growth on fly food. This strain is a useful starting point for identification of *Acetobacter* genes that are required for association with *Drosophila* and we present several potential candidates within this report. As a next step, it is important to perform mutagenesis studies on candidate genes to identify the specific bacterial factors that permit survival within a fly gut lumen. To facilitate such studies, we are developing protocols for genetic manipulation of our lab isolates of *Lactobacillus* strains. These studies are particularly important given the strain-specific effects of individual *Lactobacillus plantarum* strains on host phenotypes (Erkosar et al., 2015; Schwarzer et al., 2016; Storelli et al., 2011).

### Lactobacilli

For our studies of *Lactobacillus* genomes, we prepared whole-genome sequences of *L. brevis* or *L. plantarum* strains that we isolated from lab-raised wild-type flies, and an *L. plantarum* strain that we isolated from a wild *Drosophila*. These genomes formed the cornerstones of a comparative study that included three previously reported *Drosophila*-associated genomes (Kim et al., 2013a,b; Newell et al., 2014), and the genomes of environmental strains that successfully associate with the intestines of wild-type *Drosophila*. In this manner, we identified bacterial functions that are unique to the *Drosophila*-associated genomes of *L. brevis* and *L. plantarum* covered in this study. The functions fall into four broad categories: antibacterial, structural, metabolic, and phage-related.

The most striking feature common to all four *Drosophila*-associated *L. plantarum* genomes was the presence of broad-spectrum bactericidal factors. For example the DF, KP, WJL and DMCS_001 genomes all encode a complete *PnlMNO* operon, which encodes a bacteriocin and a corresponding immunity protein (Diep et al., 1996). Bacteriocins are produced by many lactic acid bacteria to kill neighboring bacteria, while the immunity protein protects *L. plantarum* from collateral damage (Cotter et al., 2005). In addition, the *Drosophila*-associated *L. plantarum* genomes encode the enzymatic capacity to generate 3-hydroxypropanal/reuterin, a bacterial toxin expressed by *L. reuteri* in the gut to suppress the growth of other commensals. Combined, these bactericidal molecules have the potential to counter the growth of competing bacteria inside a *Drosophila* host, and favor expansion of *L. plantarum*. The putative competitive advantages conferred by the *PnlMNO* operon and 3-hydroxypropanal may explain why *L. plantarum* is frequently reported in studies that characterize the intestinal microbiota of *Drosophila*.

The *Drosophila*-associated genomes of *L. plantarum* and *L. brevis* also encode structural components that may stabilize associations with their fly host. For example, we detected metabolic pathways for modifications to cell wells that permit host-microbe interactions and biofilm formation. These include the construction of exopolysaccharides by *L. brevis* and the regulation of sialic acid by *L. plantarum*. Sialic acid is a comparatively rare microbial metabolite, but has been observed on microbes that associate with deuterostomes. Bacteria use sialic acid as a nutrient, but they also use it to mask detection by host immune responses. While the role of sialic acid in *L. plantarum* association with *Drosophila* requires further investigation, we feel that these elements merit consideration as host-microbe interaction factors.

The *Drosophila*-associated genomes of *L. plantarum* and *brevis* also include gene products that may address nutritional requirements. Functional annotation of the respective genomes suggests that these gene products may enhance access to limited resources such as methionine by *L. plantarum* and utilization of citrate as an energy source by *L. brevis*. We were particularly struck by the presence of pectin metabolism factors within the genomes of *Drosophila*-associated strains of *L. brevis*. Pectin is an excellent source of carbon for bacteria that grow on plants; however, bacterial utilization of pectin accelerates the ripening and decay of the same plants (Abbott and Boraston, 2008). Thus, *Drosophila*-associated *L. brevis* genomes express factors that contribute to the decay of organic substrates. We consider this noteworthy, as *Drosophila* preferably consumes decayed matter as a source of nutrients. The ability of *L. brevis* to generate meals for their *Drosophila* host provides a possible explanation for the fact that *Drosophila* frequently associate with *L. brevis*. As *L. brevis* generates palatable meals for fly hosts, we speculate that their chances of association with flies in the wild are greater than those for many other bacteria. This host-microbe relationship is similar to a proposed mechanism for association of *Erwinia carotovora* with *Drosophila* in the wild (Basset et al., 2000). Our lab raised fly strains are fed a meal that contains yellow cornmeal, a potential source of pectin, possibly explaining the persistence of pectin metabolism genes in *L. brevis* strains isolated from flies.

The final difference we noted between environmental and *Drosophila*-associated *Lactobacillus* genomes was an accumulation of temperate prophage genomes throughout *Drosophila*-associated *Lactobacilli*. Intestinal stresses such as high levels of reactive oxygen species are known to trigger lysogenic induction of temperate prophages (DeMarini and Lawrence, 1992). Thus, it is feasible that bacterial strains that pass though the fly intestine will release and integrate greater numbers of lytic prophages, explaining the increased numbers of prophage genomes in *Lactobacillus* strains that associate with adult *Drosophila*.

### Acetobacter

In this study, we report the first genome of a *Drosophila*-associated strain of *A. pasteurianus*, and identified an *A. pasteurianus* strain that cannot grow on fly food. Unfortunately, our genomic comparisons are limited by the fact that only one *Drosophila*-associated genome is available for study. Nonetheless, our study yields a comparatively short list of candidate functions that may regulate growth of *Acteobacter* on nutrient medium for *Drosophila*. This list includes bacterial gene products that process nitrogen, and gene products that directly control the induction of cell death in *A. pasteurianus*. We believe that future characterization of mutations in the respective gene product has the possibility to identify the bacterial factors that control viability of *A. pasteurianus* on fly food. This approach has considerable potential given the relationship between *Acetobacter* and *Drosophila* development.

In summary, our comparative study of bacterial genomes uncovers a short list of possible genetic signatures of association with *Drosophila*. As many of the gene products have established roles in host-microbe interactions, we propose that these genes include factors that promote the frequent association of *Drosophila* with *Lactobacillus* and *Acetobacter* strains. Future characterization of mutations in the individual products will reveal the relationship between the individual factors and host physiology.

## MATERIALS AND METHODS

### Drosophila husbandry

All *Drosophila* assays were performed with virgin *w^1118^* male and female flies raised on standard corn-meal medium (Nutri-Fly Bloomington Formulation, Genesee Scientific) in a humidified incubator at 29°C. To generate germ-free flies, we transferred freshly eclosed (0-16 h old) adult flies to standard medium that we supplemented with an antibiotic cocktail (100 μg/ml ampicillin, 50 μg/ml vancomycin, 100 μg/ml neomycin and 100 μg/ml metronidazole dissolved in ethanol). This mixture has been described previously (Ryu et al., 2008). To generate gnotobiotic flies, we raised adult flies on the antibiotic cocktail for five days, starved flies for two hours, and transferred the flies to a vial containing an autoclaved fly vial cotton plug soaked with the respective bacteria. Bacterial cultures were prepared to OD600 of 50 in 5% sucrose/PBS. Twelve flies per vial were then associated with 1 ml of commensal bacteria suspension on cotton plugs. We fed the flies the bacterial meal for 16 h and transferred the flies to vials of freshly autoclaved food. Flies were raised on the initial vial for one week and transferred to fresh vials weekly thereafter. To test association, we plated fly homogenates on bacterial medium selective for *Acetobacter* (GYC agar) or *Lactobacilli* (MRS-agar) every two weeks. For *A. pasteurianus*, colony forming units were determined by independent quantification of three replicates of five flies/replicate. Flies were sterilized in 50% bleach, 75% ethanol and rinsed in water. Sterilized flies were homogenized in MRS broth (Fluka Analytical) and serial dilutions of the homogenate were plated on GYC agar plates. To test the survival of *A. pasteurianus* on fly food, bacteria were grown from for 2 days at 29°C with shaking. A bacterial culture of an OD 50 was prepared in 5% sucrose in PBS. From the OD, 50 culture serial dilutions down to 10^−7^ were prepared. 50 µl of each of the serial dilutions was added to autoclaved fly food. Vials were gently rotated to spread out the bacterial culture. Vials were plugged and incubated at 29°C for one week. Vials were rinsed with 1 ml of MRS and of the 1 ml rinse 50 µl was plated on GYC plates and incubated for 2 days at 29°C. The images shown in panels D and E of Fig. 1 correspond to the 10^−3^ dilutions.

### Bacterial isolation and sequencing

We plated homogenates of 15-day-old adult *Drosophila* on GYC and MRS culture plates. We found that *L. brevis* colonies are easily distinguished from *L. plantarum* colonies on MRS-agar medium. We isolated individual colonies of *A. pasteurianus*, *L. brevis* and the KP strain of *L. plantarum* and grew them statically at 29°C in liquid MRS (*L. brevis* and *L. plantarum*), or shaking in liquid (*A. pasteurianus*). The DF strain of *L. plantarum* was isolated from a wild, mated isofemale *Drosophila melanogaster* captured on a rotting strawberry in the kitchen of EF in Edmonton, Canada. Bacterial DNA was isolated with the Microbial DNA Isolation kit from MO BIO Laboratories Inc. (catalog number: 12224-250) according to their instructions. The genomes of *L. plantarum* strains DF and KP were sequenced and assembled at the McGill University and Génome Québec Innovation Centre on the PacBio platform. The genomes of *A. pasteurianus* (strain AD) and *L. brevis* (strain EF) were sequenced at The Applied Genomics Core of the University of Alberta. For the latter genomes, we prepared Nextera XT libraries from the isolated micribial DNA according to Illumina's protocol and sequenced the libraries with using the V3-600 cycle Kit (Illumina). Whole genome sequences were then assembled using Lasergene software (DNASTAR).

### Genome assembly and annotation

For each sequencing project, we confirmed the individual species with the SpeciesFinder 1.2 algorithm (Larsen et al., 2014) and calculated genome to genome distances with the genome to genome distance calculator of the Leibniz Institute DSMZ-German Collection of Microorganisms and Cell Cultures (Meier-Kolthoff et al., 2013). We annotated each genome with RAST (Aziz et al., 2008), identified intact prophage genomes with the PHAST server (Zhou et al., 2011) and identified CRISPR arrays with CRISPRFinder (Grissa et al., 2007). We used the CRISPRTarget algorithm to predict CRISP targets for the individual CRISPR arrays (Biswas et al., 2015). To identify regulatory proteins within the respective genomes, we used the P2RP identifier (Barakat et al., 2013). We used the GView tool to generate graphical representations of bacterial genomes (Petkau et al., 2010).
